# Supplementary material for: Orthopaedic Implant-Associated Staphylococcal Infections: A Critical Reappraisal of Unmet Clinical Needs Associated with the Implementation of the Best Antibiotic Choice
Source: Antibiotics (Basel). 2022 Mar 17;11(3):406. doi: 10.3390/antibiotics11030406 (PMC8944676; doi:10.3390/antibiotics11030406)
Supplement: Supplementary file 1 [file antibiotics-11-00406-s001.zip › antibiotics-1618389-supplementary.pdf]

antibiotics

MDPI

Perspective

Orthopaedic Implant-Associated Staphylococcal Infections: A Critical Reappraisal of Unmet Clinical Needs Associated with the Implementation of the Best Antibiotic Choice

Milo Gatti [1,2], Simona Barnini [3], Fabio Guarracino [4], Eva Maria Parisio [5], Michele Spinicci [6,7], Bruno Viaggi [8], Sara D'Arienzo [9], Silvia Forni [9], Angelo Galano [10] and Fabrizio Gemmi [9,*]

1 Department of Medical and Surgical Sciences, Alma Mater Studiorum University of Bologna, 40138 Bologna, Italy; milo.gatti2@unibo.it
2 SSD Clinical Pharmacology, Department for Integrated Infectious Risk Management, IRCCS Azienda Ospedaliero-Universitaria di Bologna, 40138 Bologna, Italy
3 Bacteriology Unit, Azienda Ospedaliero-Universitaria Pisana, 56126 Pisa, Italy; s.barnini@ao-pisa.toscana.it
4 Department of Anaesthesia and Critical Care Medicine, Azienda Ospedaliero Universitaria Pisana, 56126 Pisa, Italy; f.guarracino@ao-pisa.toscana.it
5 UOSD Microbiologia Arezzo PO San Donato–Azienda Usl Toscana Sud Est, 52100 Arezzo, Italy; evamaria.parisio@uslsudest.toscana.it
6 Department of Experimental and Clinical Medicine, University of Florence, 50134 Florence, Italy; michele.spinicci@unifi.it
7 Infectious and Tropical Diseases Unit, Careggi University Hospital, 50134 Florence, Italy
8 Neurointensive Care Unit, Department of Anesthesiology, Careggi University Hospital, 50134 Florence, Italy; bruno.viaggi@gmail.com
9 Agenzia Regionale di Sanità della Toscana, 50141 Florence, Italy; sara_88fi@hotmail.it (S.D.); silvia.forni@ars.toscana.it (S.F.)
10 SOD Microbiologia e virologia, Careggi University Hospital, 50134 Florence, Italy; galanoa@aou-careggi.toscana.it
* Correspondence: fabrizio.gemmi@ars.toscana.it

Citation: Gatti, M.; Barnini, S.; Guarracino, F.; Parisio, E.M.; Spinicci, M.; Viaggi, B.; D'Arienzo, S.; Forni, S.; Galano, A.; Gemmi, F. Orthopaedic Implant-Associated Staphylococcal Infections: A Critical Reappraisal of Unmet Clinical Needs Associated with the Implementation of the Best Antibiotic Choice. *Antibiotics* **2022**, *11*, 406. https://doi.org/10.3390/antibiotics11030406

Academic Editors: Ciro Villani and Daniele De Meo

Received: 14 February 2022
Accepted: 16 March 2022
Published: 17 March 2022

Publisher's Note: MDPI stays neutral with regard to jurisdictional claims in published maps and institutional affiliations.

Copyright: © 2022 by the author. Licensee MDPI, Basel, Switzerland. This article is an open access article distributed under the terms and conditions of the Creative Commons Attribution (CC BY) license (https://creativecommons.org/licenses/by/4.0/).

Table S1. A summary of the evidence investigating the off-label use of novel lipoglycopeptides for the management of infections associated with orthopaedic implants.

Author, year, and reference	Study design	No. of patients	Clinical features	Prior antibiotic and duration	Antibiotic and dosing	Isolates	Duration of follow-up	Outcome	Relapse rate – Resistance development	Safety (Overall proportion of AEs)
				Dalbavancin						
Morata et al., 2019 [29]	Retrospective cohort study, multicentric	64	45 Implant-associated infection<br>19 Bone or joint infection	100.0%	Dalbavancin LD 1000 mg (*n* = 50) – 1500 mg (*n* = 12) – 500 mg (*N* = 1) – 750 mg (*n* = 1)<br>Single dose (*n* = 9)<br>Followed by 500 mg/week (*n* = 54; median duration 5 weeks)<br>Followed by 1500 mg biweekly (*n* = 1; four total doses)	30 *S. epidermidis*<br>14 *S. aureus*<br>5 *E. faecalis*<br>4 *E. faecium*<br>3 *C. striatum*<br>3 *Streptococcus spp*<br>2 *S. lugdunensis*<br>1 *S. capitis*<br>1 *S. pneumoniae*	Latest medical visit	Clinical success or improvement: 97.7% (implant-associated infections)<br>Clinical success or improvement: 89.5% (bone or joint infections)<br>Mortality rate: 6.3%	Relapse: 3.1%	NA
Wunsch et al., 2019 [30]	Retrospective cohort study, multicentric	62 (101 overall patients included in the study)	32 prosthetic joint infection	100.0%	Dalbavancin 1500 mg single dose<br>or	28 CoNS<br>14 MSSA<br>8 MRSA	90 days after the last dose of dalbavancin	Overall clinical success: 89% (93.6% bone and joint infections)	NA	3% (overall)

			30 osteomyelitis<br><br>OPAT 49%		1500 mg + 1500 mg<br><br>or<br><br>1000 mg + 500 mg	7 *Enterococcus spp*<br><br>5 *Streptococcus spp*<br><br>4 *P. acnes*<br><br>21 Others		90-day mortality rate: 5%<br><br>(3.2% bone and joint infections)<br><br>Overall clinical failure: 5%<br><br>(3.2% bone and joint infections)		
Bai et al., 2020 [31]	Retrospective cohort study, multicentric	50<br><br>(82 overall patients included in the study)	25 Osteomyelitis<br><br>17 Prosthetic joint infections<br><br>4 Spondylodiscitis<br><br>4 Septic arthritis<br><br>OPAT 57.8%	82.5%	Dalbavancin 1000 mg (LD) + 500 mg<br><br>or<br><br>1500 mg single dose	28 CoNS<br><br>24 MRSA<br><br>14 MSSA<br><br>6 *E. faecalis*<br><br>5 *E. faecium*<br><br>5 Others	30-180 days	Clinical success at end of study:<br><br>89.7% (osteomyelitis – spondylodiscitis)<br><br>76.5% (prosthetic joint infections)<br><br>75.0% (septic arthritis)	Relapse:<br><br>17.8%<br><br>(overall in the study)	7.0%
Tobudic et al., 2019 [32]	Retrospective cohort study	46<br><br>(72 overall patients included in the study)	20 Osteomyelitis<br><br>14 Spondylodiscitis	81%	Dalbavancin<br><br>1000 mg LD + 500 mg weekly<br><br>1500 mg LD + 1000 mg biweekly	27 MSSA<br><br>11 *Streptococcus spp*<br><br>6 MRSA<br><br>5 MSSE	6 months	Clinical cure:<br><br>56.5%<br><br>(overall bone and joint infections)<br><br>Osteomyelitis: 60%<br><br>Spondylodiscitis: 50%	NA	8.7%<br><br>(rash *N* =2; nausea *N* =1; hyperglycaemia *N* =1)

			8 Prosthetic joint infections<br>4 Acute septic arthritis		1500 mg day 1 + 1500 mg day 8	3 MRSE<br>3 *Enterococcus spp*		Acute septic arthritis: 100%<br>Prosthetic joint infections: 38%<br>Clinical failure:<br>23.9%		
Bouza et al., 2018 [33]	Retrospective cohort study, multicentric	33	20 Prosthetic joint infection<br>12 Osteomyelitis<br>1 Septic arthritis<br>OPAT 73.9%	97.1%<br>(median 18 days)	Dalbavancin 1500 mg single dose<br>or<br>1000 mg (LD) + 500 mg	16 CoNS<br>6 MRSA<br>4 MSSA<br>3 *Enterococcus spp*<br>4 Others	NA	Clinical success:<br>84.8%	Relapse:<br>6.1%	13.0%<br>(2.9% serious)
Buzon Martin et al., 2019 [34]	Retrospective cohort study	16	All prosthetic joint infections<br>(8 total hip and 8 total knee arthroplasty infections)	NA	Dalbavancin<br>LD 1500 mg + 500 mg day 8 and then 500 mg biweekly<br>or<br>LD 1000 mg +<br>500-1000 mg/week	7 CoNS<br>4 MRSA<br>4 *E. faecium*<br>4 *E. faecalis*	503 days<br>(median)	Clinical success:<br>75%<br>Clinical failure:<br>12.5%<br>Mortality rate:<br>6.3%	Relapse:<br>12.5%	12.5%<br>(not-serious; leukopenia *N* = 1; rash *N* = 1)

Bork et *al*., 2019 [35]	Retrospective cohort study, multicentric	15<br>(28 overall patients included in the study)	13 Osteomyelitis<br>1 Prosthetic joint infection<br>1 Septic arthritis<br>OPAT 100%	100.0%<br>(median 13.5 days)	NA	8 MRSA<br>6 MSSA<br>4 CoNS<br>8 Other<br>5 NA	30-90 days	Overall clinical success:<br>71%<br>(Bone and joint infection 50% at 30-day)	NA	10.7%<br>(overall)
Brescini et *al*., 2021 [36]	Retrospective cohort study	13<br>(55 overall patients included)	13 Prosthetic joint infection	96%	Dalbavancin 1500 mg single dose<br>or<br>1500 mg + 1500 mg	9 MRSA<br>6 polymicrobial<br>3 *S. epidermidis*<br>2 *E. faecalis*<br>1 MSSA<br>1 MRSE<br>8 Others<br>25 Empirical	NA	Clinical success:<br>69.2%	NA	2%<br>(overall)
Nunez-Nunez et al., 2018 [37]	Prospective observational	10<br>(19 overall patients included in the study)	6 Osteomyelitis<br>4 Implanted prosthetic device infection	100%	Dalbavancin<br>1500 mg single dose<br>or 1500 mg + 1500 mg or 1000 mg + 500 mg	7 MRSA<br>6 CoNS<br>5 MSSA<br>1 *E. faecalis*<br>1 *E. faecium*	90 days	Clinical success:<br>100.0%	Relapse:<br>0.0%	4.5%<br>(not serious; overall)

Vazquez Deida et al., 2020 [38]	Retrospective observational case series	6	5 Osteomyelitis<br>1 Joint infection	100.0%<br>(range 28-35 days)	1500 mg single dose	3 MRSA<br>2 MSSA<br>1 GAS<br>1 MRSE	90 days	Clinical success:<br>83.3%<br>(16.7% clinical failure)	Relapse:<br>0.0%	7.0%<br>(overall)
Azamgarhi et al., 2019 [39]	Case report	1	Infected massive endoprosthetic replacement of the hip	Vancomycin + Ceftriaxone + Amikacin for 5 days	Dalbavancin<br>1500 mg for two doses	MRSE<br>Dalbavancin MIC <0.047 mg/L	16 months	Clinical success:<br>100.0%	Relapse:<br>0.0%	None
Trujillano Ruiz et al., 2019 [40]	Case report	1	Prosthetic infection of the hip	Vancomycin and then ciprofloxacin + rifampicin for 4 months, and then + Linezolid for 4 weeks	Dalbavancin<br>1000 mg LD + 500 mg/week for 3 weeks	MRSE	1 month	Clinical success:<br>100.0%	Relapse:<br>0.0%	None
Carrion Madronal et al., 2020 [41]	Case report	1	Prosthetic infection of the hip	Vancomycin for two weeks, then linezolid for two weeks	Dalbavancin<br>LD 1000 mg + 500 mg weekly for 7 weeks in combination with linezolid	MRSE	16 weeks	Clinical success:<br>100.0%	Relapse:<br>0.0%	None
Ramirez-Hidalgo et al., 2018 [42]	Case report	1	Prosthetic knee infection	Daptomycin for 10 days	Dalbavancin LD 1000 mg weeks + 500 mg/week for 3 weeks	MRSE<br>Dalbavancin MIC <0.047 mg/L	9 months	Clinical success:<br>100.0%	Relapse:<br>0.0%	None
Fernandez et al., 2016 [43]<br>Schmidt-Malan et al., 2016 [44]	In vitro study	171	Staphylococcal clinical isolates from prosthetic joint infections.<br>DAL: $MBIC_{90}$ 0.12–0.50 mg/L, $MBBC_{90}$ 2–4 mg/L<br>VAN: $MBIC_{90}$ 2–4 mg/L, $MBBC_{90}$ >128 mg/L							

			TDZ: $MBIC_{90}$ 2–4 mg/L, $MBBC_{90}$ >32 mg/L							
Knafl et al., 2017 [45]	In vitro study	20	10 MRSA plus 10 MRSE clinical strains.<br>MRSA: MIC range 0.031–0.064 mg/L; MBIC 1–4 mg/L<br>MRSE: MIC range 0.023–0.625 mg/L; MBIC 2–16 mg/L							
Neudorfer et al., 2018 [46]	In vitro study	83	25 *E. faecium* plus 58 *E. faecalis* clinical isolates.<br>DAL: for VSE: $MBIC_{90}$ 0.25 mg/L, $MBBC_{90}$ 1 mg/L for VRE: $MBIC_{90}$ > 16 mg/L, $MBBC_{90}$ >16 mg/L<br>VAN: for VSE: $MBIC_{90}$ 2 mg/L, $MBBC_{90}$ >128 mg/L for VRE: $MBIC_{90}$ > 128 mg/L, $MBBC_{90}$ > 128 mg/L<br>DAP: for VSE: $MBIC_{90}$ 4 mg/L, $MBBC_{90}$ 128 mg/L for VRE: $MBIC_{90}$ 4 mg/L, $MBBC_{90}$ 128 mg/L							
Ziemyte et al., 2020 [47]	In vitro study	Clinical isolates of MSSA, MRSA, and MRSE.<br>1. Biofilm inhibition. MBIC of DAL ranged 0.5–2 mg/L. RIF and DAL showed the highest inhibitory efficacy as compared with CLX, VAN, and LNZ.<br>2. Biofilm treatment. DAL stopped or reduced biofilm at 8–32 mg/L. Comparators had no effect for *S. aureus* biofilm. For *S. epidermidis* biofilm, RIF and CLX were more effective than DAL at lower concentrations.								
Jacob et al., 2021 [48]	In vitro study	Dalbavancin and rifampicin exhibited a concentration-dependent antibiofilm activity, with dalbavancin being more effective. At a concentration of 16 mg/l, dalbavancin reduced the viable bacteria (CFU/ml) by 2.7 log-magnitudes, while 32 mg/l of rifampicin achieved a reduction of 1.9 log-magnitudes. In combination, rifampicin increased the effectiveness of dalbavancin, indicating additive effects.								
Solon et al., 2007 [49]	Preclinical study	In a rabbit model, dalbavancin concentrations achieved a mean of 13.4 mg/L in bone marrow and a mean of 4.2 mg/L in cortical bone after a single dose of 20 mg/kg. Bone concentrations remained higher MIC_{90} for up to 336 h.								
				Oritavancin						
Redell et al., 2019 [50]	Retrospective observational multicenter	3	Prosthetic joint infections	100% of cases, including beta-lactams, clindamycin, TMP-SMX, vancomycin, and daptomycin	2/3 cases: 1200 mg single dose<br>1/3 cases: 1200 mg × 2 every 14 days	NA	NA	66.7% cured	NA	None
Schulz et al., 2017 [51]	Case series	1	Infections associated with orthopaedic implants following	Oxacillin – Daptomycin (68 days)	Oritavancin 1200 mg first dose followed by 800 mg	MSSA	NA	Clinical improvement	None	None

			laminectomy in vertebral osteomyelitis		weekly for 5 weeks					
Yan et al., 2018 [52]	In vitro study	185	67 MSSA, 37 MRSA, 22 MSSE, and 59 MRSE isolates from PJIs. The oritavancin MIC_{50} for *S. aureus* and MSSE was 0.03 μg/mL, and for MRSE, it was 0.06 μg/mL; MIC_{90} for *S. aureus* and *S. epidermidis* was 0.12 μg/mL for both the methicillin-resistant and -susceptible subgroups. The oritavancin $MBBC_{50}$ for *S. aureus* and *S. epidermidis* was 2 μg/mL for both the methicillin-resistant and -susceptible subgroups; the $MBBC_{90}$ for *S. aureus* and MSSE was 4 μg/mL, and for MRSE, it was 8 μg/mL.							
Belley et al., 2009 [53]	In vitro study	Oritavancin exhibited antibiofilm activity against MSSA, MRSA, and VRSA strains at MBECs ranging from 0.5 to 8 mg/L, providing a sterilization of the biofilm after a 1-h exposure at an MBEC of 4 mg/L. Conversely, MBECs for linezolid and vancomycin were > 128 mg/L.								
Yan et al., 2018 [54]	In vitro study	10	10 MRSA isolates from PJIs. Oritavancin combined with rifampicin demonstrated statistically significant bacterial reductions compared with those of either antimicrobial alone (rifampicin, linezolid, gentamycin) for all 10 isolates, with synergy being observed for 80% of the isolates.							
Lehoux et al., 2015 [55]	Preclinical study	In a rabbit model, bone/serum ratio of oritavancin ranged from 1.1 to 3.1 after a single dose of 20 mg/kg, with concentrations remaining stable for up to 168 h.								
				Telavancin						
Sims et al., 2021 [56]	Retrospective observational multicenter	57	30 osteomyelitis with prosthetic materials<br>27 PJIs	71.5% of cases (overall)	Median dose: 750 mg daily for a median duration of 26 days	37.8% MSRA<br>14.8% MSSA<br>10.0% CoNS	NA	75.0% clinical cure in osteomyelitis with prosthetic materials<br>84.6% clinical cure in PJIs	NA	AEs rate: 19.9% (overall)<br>Serious AEs: 2.1% (overall)
Harting et al., 2017 [57]	Case series	8	6 osteomyelitis with prosthetic implants<br>2 PJIs	37.5%	10 mg/kg/day for 9-66 days (duration range)	NA	NA	62.5% clinical cure	NA	25.0%
LaPlante et al., 2009 [58]	In vitro study	Telavancin exhibited antibiofilm activity against *Staphylococcus* and *Enterococcus* strains at MBECs below the MIC and ranging from 0.125 to 2 mg/L. Conversely, vancomycin did not demonstrate similar activity (MBECs > 128 mg/L).								

AEs: adverse events; CFU: colony format unit; CoNS: coagulase-negative Staphylococci; DAL: dalbavancin; DAP: daptomycin; GAS: group A *Streptococcus*; LD: loading dose; LNZ: linezolid; MBBCs: minimum biofilm bactericidal concentrations; MBECs: minimal biofilm eradication concentrations; MBICs: minimal biofilm inhibitory concentrations; MIC: minimum inhibitory concentration; MRSA: methicillin-resistant *S. aureus*; MRSE: methicillin-resistant *Staphylococcus epidermidis*; MSSA: methicillin-susceptible *S. aureus*; MSSE: methicillin-susceptible *Staphylococcus epidermidis*; NA: not assessed; OPAT: outpatient parental antibiotic therapy; PJIs: prosthetic joint infections; RIF: rifampicin; TDZ: tedizolid; TMP-SMX: cotrimoxazole; VAN: vancomycin; VRE: vancomycin-resistant *Enterococcus*; VRSA: vancomycin-resistant *S. aureus*; VSE: vancomycin-susceptible *Enterococcus.*

Table S2. A summary of the evidence investigating the off-label use of novel anti-staphylococcal cephalosporins for the management of infections associated with orthopaedic implants.

Author, year, and reference	Study design	No. of patients	Clinical features	Prior antibiotic and duration	Antibiotic and dosing	Isolates	Duration of follow-up	Outcome	Relapse rate – Resistance development	Safety (Overall proportion of AEs)
					Ceftaroline					
Malandain et al., 2017 [61]	Retrospective study	19	16 with infections associated with orthopaedic implants	NA	Ceftaroline 600 mg q8-12h/day for a median of 6 weeks<br><br>Combination therapy: 89.5%	11 MRSE<br><br>4 MSSE<br><br>84.2% polymicrobial	1 year	EOT: 68.4%<br><br>6 months: 36.8%<br><br>1 year: 10.5%	NA	21.1%
Park et al., 2016 [62]	In vitro study	171	97 *S. aureus* (36% oxacillin resistant) and 74 *Staphylococcus epidermidis* (74% oxacillin resistant) isolates from PJIs. Ceftaroline inhibited all staphylococci at ≤0.5 µg/mL. The ceftaroline $MIC_{90/50}$ values for MSSA, MSSE, MRSA, and MRSE were 0.19/0.125, 0.094/0.047, 0.5/0.38, and 0.38/0.19 µg/mL, respectively.							
Landini et *al*., 2015 [63]	In vitro study	Following prolonged exposure times, ceftaroline exerted a bactericidal activity both against MSSA and MRSA biofilms. However, MBECs remained above the maximum ceftaroline concentration tested (i.e., >16 mg/L) for all tested strains, resulting in lack of complete biofilm eradication.								
Barber et al., 2015 [64]	In vitro study	Ceftaroline demonstrated activity against biofilm-producing daptomycin non-susceptible MRSA. Ceftaroline plus daptomycin displayed therapeutic enhancement over monotherapy, providing a potential option for difficult-to-treat medical device infections.								
Gatin et al., 2014 [65]	Preclinical study	MRSA rabbit PJI model. No difference between ceftaroline and vancomycin in mean reduction of MRSA colonies of crushed bones was noted. Synergistic/additive activity with rifampicin was documented for both agents. None of the treatment regimens fully sterilized the bones.								
					**Ceftobiprole**					
Zhanel et al., 2021 [66]	Retrospective cohort study	9<br><br>(38 overall patients included in the study)	9 osteomyelitis and/or PJIs	100.0%	500 mg q8h<br><br>(dosage adjusted according to renal function)	8 MRSA<br><br>1 *E. faecium*	NA	Clinical cure: 77.8%<br><br>Microbiological eradication: 88.9%	NA	0.0%
Abbanat et al., 2014 [67]	In vitro study	8	6 MSSA/MRSA isolates and 2 MSSE/MRSE isolates. Relative to no-drug controls, after 7 days of exposure ceftobiprole concentrations from 1/4 *f*Cmax to *f*Cmax generally decreased CFUs in MBEC or colony biofilms of *S. aureus* isolates by ca. 1.5 log_{10} to ≥2.5 log_{10}. Following 7 days of exposure, vancomycin and rifampicin were ineffective as single agents or in combination in the colony model, but yielded CFU decreases from 0 to 5 log_{10} in the MBEC model.							
Yin et al., 2008 [68]	Preclinical study	In a rabbit MRSA osteomyelitis model, ceftobiprole achieved in the tibial matrix and marrow mean concentrations of 3.2±1.3 mg/L and of 11.2±6.5 mg/L, and of 13.4±7.3 mg/L and 66.3±43.2 mg/L, in uninfected animals treated with 20 mg/kg and 80 mg/kg of the antibiotic, respectively. Compared to vancomycin and linezolid, a significantly higher decrease in bacterial titers was reported.								

AEs: adverse events; CFUs: colony format unit; Cmax: peak concentration; EOT: end of treatment; MBBCs: minimum biofilm bactericidal concentrations; MBECs: minimal biofilm eradication concentrations; MBICs: minimal biofilm inhibitory concentrations; MIC: minimum inhibitory concentration; MRSA: methicillin-resistant *S. aureus*; MRSE: methicillin-resistant *Staphylococcus epidermidis*; MSSA: methicillin-susceptible *S. aureus*; MSSE: methicillin-susceptible *Staphylococcus epidermidis*; NA: not assessed; PJIs: prosthetic joint infections.

Table S3. A summary of the evidence investigating the off-label use of daptomycin for the management of infections associated with orthopaedic implants.

Author, year, and reference	Study design	No. of patients	Clinical features	Prior antibiotic and duration	Antibiotic and dosing	Isolates	Duration of follow-up	Outcome	Relapse rate – Resistance development	Safety (Overall proportion of AEs)
Byren et al., 2012 [69]	Randomized controlled trial	75	PJIs managed with two-stage revision	NA	Daptomycin 6 mg/kg/day (25 patients) vs. Daptomycin 8 mg/kg/day (24 patients) vs. Comparator (Vancomycin, Teicoplanin, or Semisynthetic penicillin; 26 patients)	25 MSSA 24 CoNS 13 MRSA	4 months	Clinical response: 59.6% daptomycin vs. 38.1% comparator<br>Microbiological eradication: 51.1% daptomycin vs. 38.1% comparator	NA	Discontinuation due to AEs: daptomycin 12.2% vs. 16% comparator
Carli et al., 2020 [70]	Retrospective comparative study	341	PJIs managed with DAIR or two-stage exchange	NA	Daptomycin 6-9 mg/kg/day (77 patients) vs. Other regimens	32 MSSA 22 CoNS 21 MRSA	2 years	Daptomycin use was not associated with better clinical outcome either in patients undergoing DAIR (OR 1.70; 95%CI 0.62-4.65) or two-stage exchange (OR 0.58; 95%CI 0.27-	NA	7.8%

					(cefazolin, oxacillin, or vancomycin)			1.26) when compared to other regimens.		
Joseph et al., 2019 [71]	Retrospective matched case-control study	40	PJIs	100.0%	Daptomycin 10-12 mg/kg/day (20 patients)<br>vs.<br>Vancomycin<br>15 mg/kg LD + 30-40 mg/kg/day CI (20 patients)	19 CoNS<br>13 MSSA<br>4 *E. faecalis*<br>3 *Streptococcus spp*<br>2 *P. acnes*<br>2 *Corynebacterium spp*	2 years	Clinical cure:<br>85% daptomycin vs. 90% vancomycin (p=0.63)	Relapse rate:<br>10% vs. 10% (p=0.99)	Overall AEs:<br>20% daptomycin vs. 30% vancomycin (p=0.47)<br>Discontinuation due to AEs:<br>0% vs. 25% (p=0.02)
Kuo et al., 2016 [73]	Retrospective cohort study	22	PJIs	NA	Daptomycin 6 mg/kg/day for days<br>+<br>local daptomycin in polymethylmethacrylate bone cement	10 MRSA<br>8 MRSE<br>4 CoNS	2 years	Clinical cure: 100.0%	None	4.5%
Herrera et al., 2017 [74]	Retrospective cohort study	21	PJIs<br>DAIR: 52.4%	100.0%	Daptomycin 10 mg/kg/day<br>+<br>Rifampicin 600 mg/day for 5-45 days	10 *S. epidermidis*<br>4 MRSA<br>3 MSSA<br>1 *S. lugdunensis*<br>1 *S. hominis*<br>3 Negative	NA	Clinical cure: 85.7%	NA	0.0%

Corona Perez-Cardona et al., 2012 [75]	Retrospective cohort study	20	PJIs<br>(8 cases acute<br>9 cases chronic<br>3 cases positive intraoperative culture)	100.0%	Daptomycin 6.6 mg/kg/day for a median of 44.9 days<br>Combination therapy with rifampicin in 40% of cases	8 MRSE<br>5 MSSE<br>2 MRSA<br>2 MSSA<br>2 *Enterococcus*<br>1 *P. acnes*	Median 20 months	Clinical cure: 78.6%	Relapse 21.4%	20.0%<br>(10.0% serious)
Lora-Tamayo et al., 2014 [76]	Retrospective cohort study	18	Acute PJIs due to FQ-resistant *Staphyolococci* managed with DAIR	100.0%	Daptomycin 10 mg/kg/day for 35-56 days<br>+<br>Rifampicin 600 mg/day for 35-74 days	10 MRSA<br>7 CoNS<br>1 MSSA	Up to 3 years	Clinical cure: 50%	Relapse: 28.4% of cases<br>No resistance development	0.0%
Chang et al., 2017 [77]	Retrospective cohort study	16	PJIs	56.3%	Daptomycin 8.3 mg/kg/day for a median of 14 days	10 MRSA<br>6 MRSE	2 years	Clinical cure: 87.5%	NA	6.3%
Rao et al., 2006 [72]	Prospective observational study	12	PJIs	NA	Daptomycin 4 mg/kg/day for 6 weeks	7 MRSA<br>4 MRSE<br>1 MSSA	Up to 13 months	Clinical cure: 50%	Relapse: 45%	0.0%
Yuste et al., 2014 [78]	Case report	1	PJI	Vancomycin, Levofloxacin, Ampicillin + Ceftriaxone, Linezolid	Daptomycin 10 mg/kg/day for 12 weeks	*E. faecalis*	39 months	Clinical cure	No	None
Luengo et al., 2018 [79]	Case report	1	Difficult-to-treat infection of a total femoral	Ampicillin + Ceftriaxone, then Amoxicillin and Vancomycin	Daptomycin 10 mg/kg/day for 42 days<br>+	*E. faecalis*	2 years	Clinical cure	No	None

			replacement	Fosfomycin 2 g q6h/day for 42 days	
Montange et al., 2014 [80]	Phase I study	16	Mean daptomycin bone penetration rate of 14.1±11.9% in 16 arthroplasty patients after 8 mg/kg dose with mean concentrations of 36.3±2.9, 6.5±3.6, and 21.6±6.8 mg/mL in shinbone, thighbone, and synovial fluid, respectively.		
Molina Manso et al., 2013 [81]	In vitro study	32	17 *S. aureus* and 15 *S. epidermidis* isolates from PJIs. Daptomycin was not effective against biofilms in either species, with minimum biofilm eradication concentrations (MBECs) significantly above the minimum inhibitory concentrations (>1024 mg/L).		
Garrigos et al., 2010 [82]	In vitro/animal study	Only daptomycin (at 6 mg/kg and 10 mg/kg) alone and in combination with rifampicin achieved a bactericidal effect in log and stationary phases against MRSA compared to vancomycin and linezolid. Furthermore, in a rat foreign-body infection model, a therapeutic regimen with daptomycin at 100 mg/kg/day for 7 days performed better than vancomycin and linezolid. In combination with rifampin, both dosages of daptomycin (100 mg/kg/day and 45 mg/kg/day) were significantly better than all other combinations, but daptomycin at 100 mg/kg/day plus rifampin achieved better cure rates at day 11 (P < 0.05) than daptomycin at 45 mg/kg/day plus rifampin. Resistant strains were found in monotherapies with rifampin and daptomycin at 45 mg/kg/day.			
Jahanbakhsh et al., 2020 [83]	In vitro study	Combination therapy including daptomycin at a dosage of 8-14 mg/kg and ceftaroline or rifampicin provides promising results in infections caused by biofilm producing VRE.			

AEs: adverse events; CFU: colony format unit; CI: continuous infusion; CoNS: coagulase-negative *Staphylococci*; DAIR: debridement and implant retention; FQ: fluoroquinolone; LD: loading dose; MBBCs: minimum biofilm bactericidal concentrations; MBECs: minimal biofilm eradication concentrations; MBICs: minimal biofilm inhibitory concentrations; MIC: minimum inhibitory concentration; MRSA: methicillin-resistant *S. aureus*; MRSE: methicillin-resistant *Staphylococcus epidermidis*; MSSA: methicillin-susceptible *S. aureus*; MSSE: methicillin-susceptible *Staphylococcus epidermidis*; NA: not assessed; OR: odds ratio; PJIs: prosthetic joint infections; VRE: vancomycin-resistant *Enterococcus.*

Table S4. A summary of the evidence investigating the off-label use of linezolid and tedizolid for the management of infections associated with orthopaedic implants.

Author, year, and reference	Study design	No. of patients	Clinical features	Prior antibiotic and duration	Antibiotic and dosing	Isolates	Duration of follow-up	Outcome	Relapse rate – Resistance development	Safety (Overall proportion of AEs)
Soriano et al., 2007 [84]	Prospective observational study	85	69 PJIs<br>All were orthopaedic implant-associated infections	NA	Linezolid 600 mg q12h for a median of 60 days	45 MRSE<br>9 MRSA	> 12 months	Remission rate: 68.2%	NA	10.5%
Gomez et al., 2011 [85]	Prospective observational study	49	PJIs	100.0%	Linezolid<br>600 mg q12h<br>+<br>Rifampicin<br>300 mg q12h<br>for a mean of 80.2 days	22 MRSE<br>6 MRSA	2 years	Remission rate: 69.4%	NA	36.7%
Morata et al., 2014 [90]	Retrospective cohort study	39	Acute PJIs managed with DAIR	NA	Linezolid 600 mg q12h for a median of 44.5 days<br>±<br>Rifampicin<br>(in 22 patients)	23 MRSE<br>10 MSSE<br>5 MSRA<br>4 MSSA<br>4 *E. faecalis*<br>2 *S. viridans*	Median 2.5 years	Clinical cure: 71.8%<br>No difference in outcome between patients receiving concomitant therapy with or without rifampicin	Relapse: 20.5%	38%
Senneville et al., 2006 [91]	Retrospective cohort study	37<br>(overall 66 patients included in the study)	37 orthopaedic device-related infections	NA	Linezolid 600 mg q12h for a median of 13 weeks	68.1% MRSA	> 12 months	Clinical remission: 81%	Relapse: 10.8%	54.5%

			of which 27 PJIs							
Rao et al., 2007 [86]	Prospective observational study	34 (overall 53 patients included in the study)	34 orthopaedic device-related infections of which 23 PJIs	NA	Linezolid 600 mg q12h for a mean of 9.6 weeks	21 MRSA<br>17 MRSE<br>6 MSSA<br>5 VRE<br>2 MSSE<br>2 other	Mean 24.9 months	Clinical cure: 91.2%	Relapse: 2.9%	22.6%<br>Discontinuation due to serious AEs: 5.7%
Cobo et al., 2013 [87]	Prospective observational study	25	Late-chronic PJIs managed with two-step exchange	NA	Linezolid 600 mg q12h for 6 weeks	18 CoNS<br>4 *S. aureus*<br>3 *Streptococcus spp*<br>1 *P. acnes*<br>1 *C. striatum*	1 year	Clinical improvement: 91%<br>Microbiological eradication: 86%	Relapse: 9%	76% (overall)<br>Discontinuation due to serious AEs: 12%
Bassetti et al., 2005 [92]	Retrospective cohort study	20	PJIs	75%	Linezolid 600 mg q12 h ev/os for a mean of 7.2 weeks	14 MRSA<br>5 MRSE<br>1 *Enterococcus spp*	12 months	Clinical cure: 80%	Relapse: 20%	No cases of treatment discontinuation due to AEs
Razonable et al., 2004 [93]	Retrospective cohort study	20	75% orthopaedic implant-associated infections	NA	Linezolid 600 mg q12h for a range of 3-60 weeks	9 MRSE<br>3 MRSA	Median 276 days	Clinical cure or improvement: 90%	Relapse: 5%	55%
Nguyen et al., 2009 [88]	Retrospective matched case-control study	18 (overall 28 patients included in the study)	Orthopaedic implant-associated infections of which 11 PJIs	NA	Linezolid 600 mg q12h for a range of 2-60 weeks<br>+<br>Rifampicin	NA	3-62 months	Clinical remission: 94.1%<br>Overall no difference in clinical cure between the two	NA	42.9% of which discontinuation occurred in 14.3% of patients<br>No differences between the two therapeutic regimens in terms

					vs. TMP/SMX + Rifampicin			therapeutic regimens (89.3% vs. 78.6%; p=0.47)		of overall AE rate: 42.9% vs. 46.4% (p=0.99)
Vercillo et al., 2007 [94]	Retrospective cohort study	14	Orthopaedic implant-associated infections of which 4 PJIs	NA	Linezolid 600 mg q12h for 6 weeks	10 MRSA	> 6 months	Clinical remission: 100.0%	None	0%
Oussedik et al., 2008 [95]	Retrospective cohort study	14	Infected total joint arthroplasty treated by 1 or 2-stage revision	NA	Linezolid 600 mg q12h for 3-12 weeks	4 MRSA 4 MRSE	9-62 months	Clinical remission: 100.0%	None	21.3%
Papadopoulos et al., [89]	Retrospective matched case-control study	10 (overall 51 patients included in the study)	10 orthopaedic device-related infections of which 8 PJIs	NA	Linezolid 600 mg q12h for 2-13 weeks	4 MRSE 3 MRSA	Up to end of treatment	Clinical remission: 80.0%		44% vs. 6% in control group
Benavent et al., 2021 [96]	Retrospective multicenter study	29 (overall 51 patients included in the study)	29 orthopaedic device-related infections of which 17 PJIs	NA	Tedizolid 200 mg/day for a median of 29 days	26 CoNS 11 MSSA 10 MRSA 13 Others	Median 630 days	Clinical cure in orthopaedic device-related infections: 83% Clinical cure in PJIs: 76% No difference between	Failure in 24% of PJIs and in 17% of cases of orthopedic device-related infections	6% (mild)

								monotherapy and combination therapy with rifampicin		
Ferry et al., 2018 [97]	Case report	1	PJI	Daptomycin + Ceftaroline, Linezolid	Tedizolid 200 mg/day for long-term suppressive therapy	*S. epidermidis*	4 months	Clinical cure 100.0%	None	None
Rana et al., 2002 [98]	Phase I study	Ten patients undergoing prosthetic joint replacement were enrolled, and linezolid 600 mg q12h orally was administered. Mean concentrations in cancellous bone, synovium, and synovial fluid were >4 mg/mL that are at least double the MIC_{90} for both *Staphylococci* (2 mg/L) and *Streptococci* (1 mg/L).								
Kutscha-Lissberg et al., 2003 [99]	Phase I study	Bone and joint penetration of linezolid 600 mg was assessed in 13 patients suffering from implant-associated infections with methicillin-resistant *staphylococci.* Mean concentrations of linezolid in infected tissues were greater than 10 mg/liter in a sampling time range of 35 to 124 min after administration of the preoperative dose, except in bone specimens, where they reached 3.9 +/- 2.0 mg/liter.								
Abad et al., 2019 [100]	In vitro study	3	Three MRSA strains retrieved from bone and joint infections. Linezolid and tedizolid weakly reduced the intracellular inoculum of *S. aureus* in a strain-dependent manner despite the similar MICs for the tested strains. Additionally, both agents were ineffective in eradicating mature biofilm formed in vitro, with MBEC >2000 and >675 mg/L, respectively, for linezolid and tedizolid. However, linezolid, and in particularly tedizolid, are able to prevent biofilm formation.							
El Haj et al., 2018 [101]	In vitro study	2	One MRSA and one MSSA biofilm-producing strains were assessed. TMP/SMX and linezolid alone showed no and low activity, respectively, but no resistance emerged. The combinations with rifampicin significantly increased the anti-biofilm efficacy against MSSA (Δlog cfu/mL 56h-0h: TMP/SMX-RIF: -2.9 and LDZ-RIF: -3.1), but rifampicin-resistant strains appeared with TMP/SMX-RIF. Against MRSA, LZD-RIF (-3.1) protected against the emergence of resistance and was more effective than the TMP/SMX-RIF combination (-0.6, $p<0.05$), in which RIF-resistant strains were again detected. LVX-RIF combination confirmed its high efficacy against biofilm-embedded bacteria, this being the most effective therapy (-5.1 against MSSA).							

AEs: adverse events; CoNS: coagulase-negative *Staphylococci*; LVX: levofloxacin; MBBCs: minimum biofilm bactericidal concentrations; MBECs: minimal biofilm eradication concentrations; MBICs: minimal biofilm inhibitory concentrations; MIC: minimum inhibitory concentration; MRSA: methicillin-resistant *S. aureus*; MRSE: methicillin-resistant *Staphylococcus epidermidis*; MSSA: methicillin-susceptible *S. aureus*; MSSE: methicillin-susceptible *Staphylococcus epidermidis*; NA: not assessed; PJIs: prosthetic joint infections; RIF: rifampicin; TMP-SMX: cotrimoxazole; VRE: vancomycin-resistant *Enterococcus.*

Table S5. A summary of the evidence investigating the off-label use of fluoroquinolones for the management of infections associated with orthopaedic implants.

Author, year, and reference	Study design	No. of patients	Clinical features	Prior antibiotic and duration	Antibiotic and dosing	Isolates	Duration of follow-up	Outcome	Relapse rate – Resistance development	Safety (Overall proportion of AEs)
Lora-Tamayo et al., 2016 [122]	Randomized controlled trial	63	PJIs managed with DAIR	NA	Levofloxacin 750 mg/day + Rifampicin 600 mg/day for 8 weeks (33 patients) vs. 3-6 months (30 patients)	All caused by *S. aureus* or CoNS isolates	Median 540 days	Cure rate: 58% vs. 73% (long vs. short schedules; ITT analysis; difference −15.7%, 95% CI −39.2% to 7.8%) Cure rate: 95.0% vs. 91.7% (long vs. short schedules: per-protocol analysis; difference 3.3%, 95% CI −11.7% to 18.3%)	Relapse rate: 7%	15.9%
Nguyen et al., 2015 [123]	Retrospective cohort study	154	PJIs (35.1% DAIR)	100.0%	Levofloxacin 750 mg/day + Rifampicin 600 mg q12h/day	74 CoNS 60 MSSA 19 *Streptococcus spp* 7 *P. acnes* 3 *Corynebacterium spp*	Mean 55.6 months	Clinical remission rate: 82.5%	Clinical failure: 17.5%	31.2% (rifampicin) 8.4% (levofloxacin)
Wouthuyzen-Bakker et al., 2018 [124]	Retrospective case-control study	58	PJIs managed with DAIR	100.0%	Levofloxacin 500 mg/day +	All caused by MSSA	Mean 50-67 months	Clinical cure rate: 89% vs 87.5% (p=0.89)	Relapse rate: 5.2%	NA

					Rifampicin<br>600 mg/day<br>(40 patients)<br>vs.<br>Moxifloxacin<br>400 mg/day<br>+<br>Rifampicin<br>450 mg/day<br>(18 patients)					
Fily et al., 2019 [125]	Retrospective cohort study	23	PJIs managed with DAIR, or 1- or 2-stage exchange	87%	Moxifloxacin<br>400 mg/day<br>+<br>Rifampicin<br>median<br>11 mg/kg/day	12 *Streptococcus spp*<br>6 *P. acnes*<br>5 *E. faecalis*	Up to 44 months	Clinical cure rate: 78.3%	Relapse: 4.3%	30.4%
Muller-Serieys et al., 2009 [119]	Preclinical study	In a rabbit model of prosthetic knee infection due to a susceptible clinical strain of *S. aureus*, the combination of levofloxacin and rifampicin was bactericidal, significantly reduced bacterial titers in bone compared with levels for rifampicin and controls ($p < 0.05$) and prevented the selection of resistant mutants that was observed with rifampicin alone.								
Melendez-Carmona et al., 2019 [126]	In vitro study	10	Ten MSSA strains for infection in an osteoblastic cell model receiving treatment with levofloxacin and rifampicin. Levofloxacin was the most effective treatment at both cortical and cancellous bone concentrations (-2.4 to -1.9 log 10 CFU, respectively). The addition of rifampicin to levofloxacin did not improve performance (-1.9 log 10 CFU for cortical concentration and -1.8 log 10 CFU for cancellous concentration). An increase in small colony variants was observed in the presence of rifampicin. The addition of rifampicin to levofloxacin showed no benefit, but could account for an increased number of small colony variants.							

AEs: adverse events; CFU: colony format unit; CoNS: coagulase-negative *Staphylococci*; DAIR: debridement and implant retention; ITT: intention-to-treat; LD: loading dose; MSSA: methicillin-susceptible *S. aureus*; NA: not assessed; PJIs: prosthetic joint infections.
